# Comparative Evaluation of Volatile Organic Compounds in Two Bottle Gourd Accessions with Distinct Fruit Shapes

**DOI:** 10.3390/foods12213921

**Published:** 2023-10-26

**Authors:** Bazgha Zia, Bidisha Chanda, Jinhe Bai, Andrea Gilliard, Kai-Shu Ling

**Affiliations:** 1 U.S. Vegetable Laboratory, United States Department of Agriculture-Agricultural Research Service, Charleston, SC 29414, USA; bazgha.zia@usda.gov (B.Z.); bidisha.chanda@gmail.com (B.C.); andrea.gilliard@usda.gov (A.G.); 2 Horticultural Research Laboratory, United States Department of Agriculture-Agricultural Research Service, Fort Pierce, FL 34945, USA; jinhe.bai@usda.gov

**Keywords:** bottle gourd, *Lagenaria siceraria*, plant disease, health care GC-MS, volatile organic compounds

## Abstract

Bottle gourd (*Lagenaria siceraria* L.) belongs to the cucurbit family and has a long history of cultivation in tropical and subtropical regions worldwide, both for food and medicine. Popularized by its unique fruit shapes, gourds are used to make ornaments and musical instruments. However, there is limited information on volatile organic compounds (VOCs) in the bottle gourd fruit. In the present study, we conducted a comparative analysis of VOCs profiled in two accessions (USVL5 and USVL10) with distinct fruit shapes: bottle and cylinder. While USVL5 only produced long cylinder fruits, USVL10 produced two fruit types, cylinder (USVL10CYN) and bottle (USVL10A and USVL10B). VOCs in each line were analyzed using headspace solid-phase microextraction–gas chromatography/mass spectrometry (HS-SPME-GC/MS). Aliphatic aldehydes and alcohols were the most abundant compounds found in these bottle gourd accessions. Based on the functional profile of the identified VOCs, our results reveal the suitability of our tested line (USVL10), enriched in functionally important VOCs such as hexanal (abundance = 381.07), nonanal (abundance = 9.85), 2-methoxy-2-methylpropane (abundance = 21.26) and D-limonene (abundance = 31.48). The VOCs profiling and functional analyses support the notion that the bottle gourd accession USVL10 can be a good candidate for its use in agriculture, the health care industry and domestic uses.

## 1. Introduction

Bottle gourd (*Lagenaria siceraria* L.) (2n = 2x = 22) originated in sub-Saharan Africa, belongs to the family *Cucurbitaceae* [1] and is consumed as a vegetable worldwide, but predominantly in Asian countries [1]. Its fruit is characterized by having a yellowish green rind and a whiter pulp [2], with a diverse fruit shape including round, pyriform, hulu (double-gourd), slender straight, corbel, long handled, round and tubby [3,4]. Bottle gourd fruit is used for food consumption and for medicinal and decorative purposes. Additionally, the bottle gourd plant provides an excellent source of rootstock to the other cucurbit crops, contributing to disease resistance and cold tolerance [5,6,7].

The nutritional profile of bottle gourd is well known for its popularity for use in culinary practices, making it valuable for human health studies, and its medicinal properties, particularly in ayurvedic studies and applications [8]. The leaves and flowers of the crop have also been shown to have important medicinal applications [8].

Bottle gourd consumption has been shown to have several health benefits and functional properties, including antihyperlipidemic activity [8], antioxidant activity [9], diuretic activity, analgesic and anti-inflammatory activity [10], immuno-modulatory activity, hepatoprotective activity, cardioprotective activity, antidiabetic activity, central nervous system activity, hypertensive activity, anticancer activity and central nervous system (CNS) depressant activity [10].

Bottle gourd juice has been proven to effectively treat human diseases and is advocated in the treatment of diabetes, hypertension, flatulence, liver diseases, weight loss, painful teeth and gum ulcers [11]. It is also used to treat anasarca ascites and beri-beri. Its anti-swelling properties are useful in treating abdominal swelling and swelling of the feet as well. It is also known for its cooling properties and is widely used in Ayruveda (the ancient Indian medical system) to treat various ailments.

Volatile organic compounds play a major role in determining the aroma, flavor and medicinal properties of bottle gourd fruit. To dissect the basis of the remarkable health benefits of bottle gourd, it is important to study the VOC profile of the bottle gourd fruit for its use in the food industry, health care industry and diagnostic research sector, as well as in plant disease management [6]. Volatile organic compounds (VOCs) have gained attention as important modulators of the human health care industry, the plant health industry and the food industry. Various studies have previously been conducted in other crops, such as cucumber, to evaluate their volatile organic compounds. A total of twenty cucumber lines were used to assess the flavor-related VOCs, resulting in the identification of a Korean line showing potentially less intensive flavor in comparison to other cucumber lines on the basis of the appearance of distinct flavor-related VOCs [12]. Other cucurbit crops similar to cucumber have been widely studied and evaluated for their volatile organic compound profile with respect to flavor [13] and aroma [14]. Similarly, some studies have been conducted in watermelon to assess the outcomes of grafting in commercial bottle gourd rootstock. Guler et al. (2014) tested the potential of bottle gourd as a rootstock using the VOC profile and concluded that the local bottle gourd accessions were promising to be used for rootstock based on the appearance of favorable VOCs [5,11]. Similarly, with the growing potential of bottle gourd for health and nutritive benefits, Akad et al. recently compared the metabolomic profiles of bottle gourd and cucumber, resulting in bottle gourd being ranked higher than cucumber in terms of metabolite profile [15]. Bottle gourd has been largely explored for its use in the food industry, based on its flavor [16] and aroma profile [17], and in the health care industry [10], due to its remarkable medicinal properties. However, a comprehensive study of the VOC profile solely comparing the various bottle gourd accessions has not been reported yet. Extremely limited knowledge has been gained regarding its use in the agriculture sector, particularly in the management of plant diseases. Therefore, it is important to evaluate various accessions of bottle gourd to assess the suitability of bottle gourd alone to gain the maximum benefits of its remarkable properties for use in various fields of research and diagnostics. Keeping in mind the beneficial components of bottle gourd fruit use in the food industry, plant health managment industry, human health care industry, and diagnostics and research sector, our study primarily focusses on assessing the VOC profile of two different bottle gourd accessions and their derivatives, USVL5 and USVL10 (USVL10 with three fruit types, USVL10CYN, USVL10A and USVL10B).

The objectives of our study are to evaluate and compare the potential of the bottle gourd accessions, and to comprehensively screen and identify the best candidate for its potential benefits in the agriculture industry and health care industry, based on the appearance of its volatile organic compounds. Thus, this study deploys GC-MS for studying the volatile compound profile during the green stage of the fruits in order to assess and identify the most suitable accession to be deployed further in plant breeding programs for enhancing the plant health sector of agriculture, the human health care industry and the domestic uses of bottle gourd.

## 2. Materials and Methods

### 2.1. Plant Material, Chemicals and Reagents

**Figure 1:**
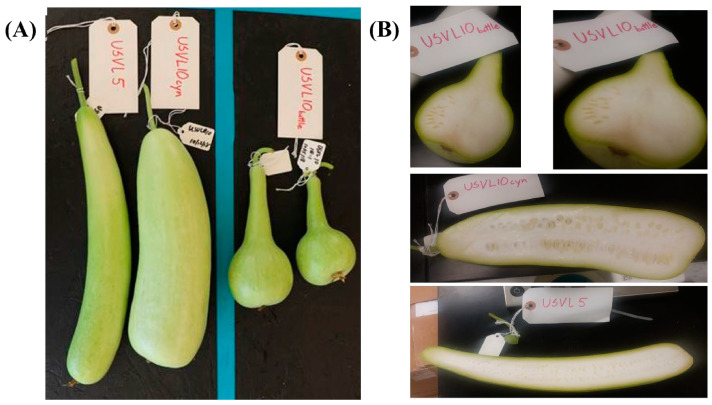
Variation in fruit shapes among the tested bottle gourd accessions. (**A**) The bottle gourd accessions shown here vary in shape, with two accessions, USVL5 and USVL10CYN, having a cylindrical shape and USVL10A and USVL10B showing bottle shapes. (**B**) Transverse section of bottle gourd fruit for USVL5, USVL10CYN and USVL10.

Two bottle gourd (*L. siceraria*) accessions, USVL5 and USVL10, were grown in a greenhouse under standard conditions. USVL5 was derived from PI 381834, while USVL10A, USVL10B and USVL10CYN were derived from a single line USVL10 (originally derived from PI 181948). USVL5 has elongated fruits with virus resistance, while USVL10CYN (elongated shape), USVL10A and USVL10B accessions with segregating fruit shapes (bottle and cylinder shapes) (Figure 1) are susceptible to virus infection.

The fruits were harvested during the green stage and processed for VOC determination using GC-MS. Volatile standard compounds (Table 1) and n-alkane standards for retention indices (RIs) were purchased from Sigma Aldrich (St. Louis, MO, USA). High-performance liquid-chromatography-grade dichloromethane (DCM), water and sodium chloride were purchased from Sigma Aldrich (St. Louis, MO, USA) at the Horticultural Research Laboratory, Fort Pierce, FL, USA.

### 2.2. Sample Preparation and Determination of VOCs 

The VOCs were analyzed using a headspace GC–MS system, as previously reported [18]. Briefly, sampling was performed by cutting the fruit flesh into 5 mm cubes using a sharp stainless-steel knife, immersing it in liquid nitrogen, and grinding it into a powder using a mortar and pestle. A total of 4 g frozen sample, together with 2 mL saturated sodium chloride solution, was transferred to a 20 mL vial and sealed with Teflon-lined septa, and then stored at −80 °C until analysis. For the sample incubation, headspace volatile extraction and injection, an autosampler (Model MPS2; Gerstel Inc., Linthicum, MD, USA) equipped with a cooled tray holder (a cooling plate (Laird Technologies, Earth City, MO, USA) controlled using a Peltier thermostat (CTC Analytics AG, Zwingen, Switzerland)) was used. Vials with tissue homogenizer were held for 0 to 4 h at 4 °C in the cooled tray. The samples were incubated for 30 min at 40 °C. A 2 cm solid-phase microextraction (SPME) fiber (50/30 μm DVB/Carboxen/PDMS; Supelco, Bellefonte, PA, USA) was exposed to the headspace for 30 min at 40 °C. After exposure, the SPME fiber was inserted into the injector of a GC-MS (Model 6890; Agilent Technologies, Santa Clara, CA, USA) to desorb the extract for 15 min at 250 °C. A 2 cm tri-phase SPME fiber (50/30 μm DVB/Carboxen/PDMS, Supelco, Bellefonte, PA, USA) was exposed to the headspace for 30 min at 40 °C to collect and concentrate volatiles prior to insertion into the injector of a DB-5 column (60 m × 0.25 mm i.d., 1.00 µm film thickness, J&W Scientific, Folsom, CA, USA) installed in an Agilent 7890 GC coupled with a 5975 MS detector (Agilent Technologies, Palo Alto, CA, USA). Compounds were tentatively identified by matching their mass spectra to entries in the NIST 14 (https://www.nist.gov/srd/nist-special-database-14, National Institute of Standards and Technology, Gaithersburg, MD, USA) library and the authentic volatile compound standards, as well as by comparing their RIs with corresponding literature data [19]. For quantification, the peak size (total ion current) was used to compare the relative abundance between samples.

### 2.3. Statistical Analysis

All of the data for all of the VOCs across the two lines and their derivatives were recorded in triplicate and subjected to ANOVA at the 0.05 level of significance using the R code. Based on the results, there was no need to conduct post hoc tests. Descriptive statistics were used to calculate means and these were compared using MS Excel to determine statistically significant differences between the identified VOCs for each line. Additionally, a heatmap was plotted using the heatmaply 1.4.2 R package (https://cran.r-project.org/package=heatmaply) and the clustering distance functions in R [20]. The separation of the VOCs identified in the four bottle gourd accessions was analyzed using principal component analysis in R [21]. 

## 3. Results

### 3.1. VOC Profile of the Tested Bottle Gourd Accessions and Derivatives 

GC-MS analysis in combination with headspace-SPME and a comparison of the mass spectra with the NIST 14 library resulted in the identification of 60 VOCs in the four bottle gourd accessions. The analysis of variance showed that there was not a significant difference between the tested lines and the variable abundance, with F = 0.67 and *p* = 0.572. The identified VOCs belonged to the following categories: aliphatic alcohols (twelve), aliphatic aldehydes (fourteen), aliphatic ketones (five), amines (two), aliphatic alkanes (eleven), aliphatic alkenes (two), aromatic compounds (four), monoterpenoids (three), furans (four) and other VOCs (four) (Table 1).

**Figure 2:**
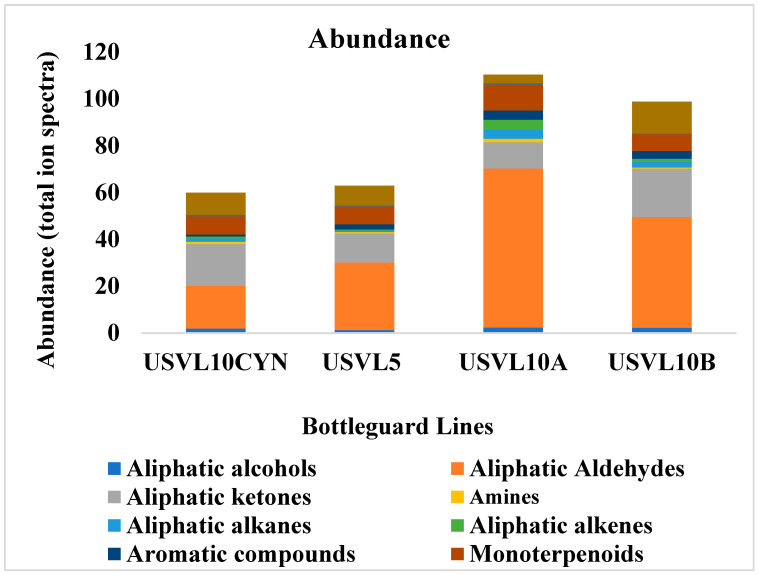
Abundance (total ion spectra) of compositions of the major classes of volatile organic compounds (VOCs) in the fruit of the tested bottle gourd lines, USVL10CYN, USVL5, USVL10A and USVL10B.

**Figure 3:**
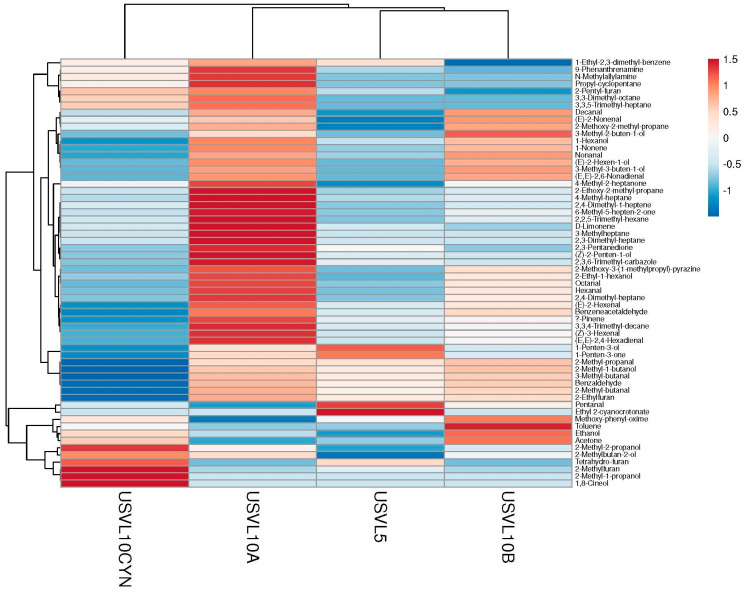
Cluster analysis and heatmap of investigated VOCs in the tested bottle gourd lines.

Among them, the aliphatic aldehydes constituted a major part of the identified VOCs, with an RT ranging from 12.09 to 39.52 (Table 1, Figure 2 and Figure 3). The aldehydes were the leading group, consisting of decanal 3-methyl-butanal, 2-methyl-butanal, pentanal, (Z)-3-hexenal, hexanal, (E)-2-hexenal, (E,E)-2,4-hexadienal, octanal nonanal, (E,E)-2,6-nonadienal, (E)-2-nonenal and 2-methyl-propanal (Table 1). Similarly, the aliphatic ketones consisted of a total of five compounds, including acetone, 1-penten-3-one, 2,3-pentanedione, 4-methyl-2-heptanone and 6-methyl-5-hepten-2-one (Table 1). The aliphatic alcohols consisted of a total of twelve compounds, including ethanol, 2-methyl-2-propanol, 2-methyl-1-propanol, 1-penten-3-ol, 3-methyl-3-buten-1-ol, 2-methyl-1-butanol, (Z)-2-penten-1-ol, 3-methyl-2-buten-1-ol, (E)-2-hexen-1-ol, 1-hexanol, 2-ethyl-1-hexanol and 2-methylbutan-2-ol (Table 1). Similarly, the aliphatic alkanes consisted of a total of 11 VOCs, propyl-cyclopentane, 2-methoxy-2-methylpropane, 2-ethoxy-2-methyl propane, 3,3,4-trimethyl-decane, 4-methyl-heptane, 3-methylheptane, 2,3-dimethyl-heptane, 2,4-dimethyl-heptane, 3,3,5-trimethyl-heptane,2,2,5-trimethyl-hexane, 2,4-dimethyl-1-heptene and 3,3-dimethyl-octane (Table 1). The aliphatic alkenes consisted of only two compounds, 1-nonene and 2,4-dimethyl-1-heptene (Table 1). Similarly, the amines also included two compounds, 9-phenanthrenamine and N-methylallylamine. The aromatic compounds constituted a total of four compounds, including benzaldehyde, ben eneacetaldehyde, 1-ethyl-2,3-dimethyl-benzene and toluene (Table 1). The monoterpenoids consisted of three compounds 1,8-cineol Eucalyptol, D-limonene and α-pinene. A total of four compounds constituted the furans, including 2-Methylfuran, Tetrahydro-furan, 2-pentyl-furan and 2-ethylfuran. Other miscellaneous compounds (2-methoxy-3-(1-methylpropyl)-pyrazine, Methoxy-phenyl-oxime, ethyl 2-cyanocrotonate and 2,3,6-trimethyl-carbazole) were also found to contribute to the VOC profile of bottle gourd accessions (Table 1).

### 3.2. Ranking of Bottle Gourd Accessions for Identified VOCs

Among the two bottle gourd accessions and their derivatives with varied fruit shapes, the highest abundance of aliphatic aldehydes, alcohols, alkanes, alkenes, amines, aromatic compounds and monoterpenoids was noted for USVL10A (Figure 2 and Figure 3). The aliphatic aldehydes dominated the USVL10A profile. Similarly, USVL10B showed the second highest percentage of aliphatic alcohols, aldehydes, alkanes, alkenes and aromatic compounds and the highest percentage of aliphatic ketones and other compounds (Figure 2). USVL10CYN consisted of the highest percentage of furans. On the other hand, USVL5 showed a comparatively lower abundance of VOCs dominated by aliphatic aldehydes, aliphatic ketones, monoterpenoids and other miscellaneous VOCs. Overall, the derivated line of USVL10 (USVL10A, USVL10B and USVCYN) has the highest percentage of prominent VOCs in comparison to USVL5 (Figure 2). 

**Figure 4:**
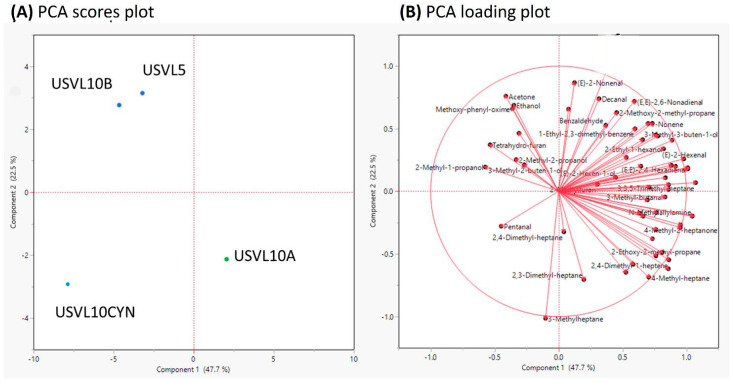
Principal component analysis (PCA) of 60 important volatile compounds measured in the fruits of the tested bottle gourd accessions. (**A**) PCA scores plot and, (**B**) PCA loadings plot.

The PCA also showed that USVL10A depicts a distinct blend of VOCs in comparison to the other three bottle gourd accessions and derivates (USVL10CYN, USVL5, USVL10B) (Figure 4A). PC1 with a variance of 47.7% and PC2 with a variance of 22.5% of the total variance was detected among the tested samples (Figure 4A). All the 60 VOCs were detected in all the tested bottle gourd accessions, respectively (Figure 4B).

### 3.3. Functional Profiling of Identified VOCs among the Tested Accessions

**Figure 5:**
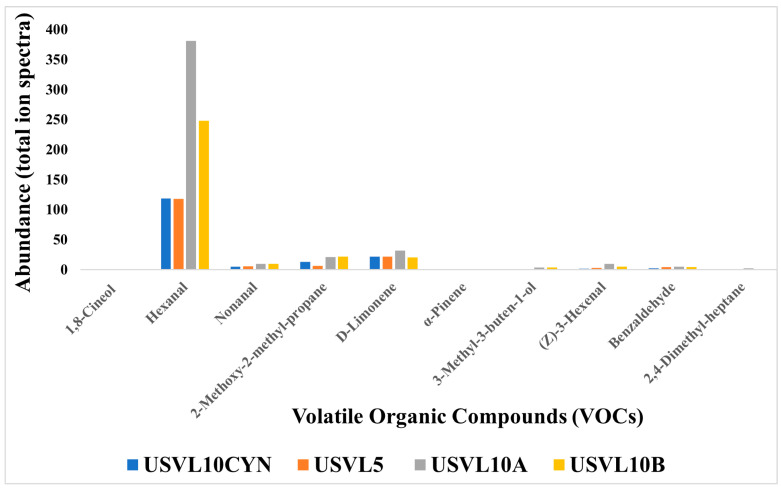
Abundance (total ion spectra) compositions of the volatile organic compounds (VOCs) with significant uses in food industry, plant disease health management and human disease diagnostics and research sector, in the tested bottle gourd accessions, USVL10CYN, USVL5, USVL10A and USVL10B.

Based on the functional characteristics, a total of nine compounds were found to be important for various purposes. These compounds consisted of hexanal, nonanal, 2-methoxy-2-methylpropane, D-limonene, α-pinene, 3-methyl-3-buten-1-ol-, (z)-3-hexenal, hexanal, benzaldehyde, 2,4-dimethyl-heptane and 1,8-cineol (Figure 5). These functionally important VOCs play an important role in plant disease management and also contribute to the health care industry. Among our tested accessions, USVL10A showed a higher abundance ratio for hexanal, nonanal, 2-methoxy-2-methylpropane and D-limonene. USVL10B is ranked second for the appearance of hexanal, nonanal, 2-methoxy-2-methylpropane, D-limonene in our study. However, we will consider USVL10A to be the most prominent candidate for its suitability to be used for plant disease management in agriculture and health care industry.

## 4. Discussion

Understanding the volatile organic compounds in different accessions of bottle gourd is important to comprehensively address the potential of bottle gourd fruit, adding value to its industrial use in medicine, domestic purposes, agriculture, and health care industry. Previously bottle gourd has been evaluated for VOCs as a source of grafting, or in combination with other fruits and vegetables [5,11]. Here we are evaluating only the bottle guard accessions to comprehensively profile the appearance of VOCs naturally occurring in two different bottle gourd accessions and their derivatives. 

Among the two bottle gourd accessions and the derivates tested in this study, a total of 60 VOCs were identified (Table 1). Many identified VOCs belonged to the aliphatic aldehydes, alcohols and alkanes group. We found the chemical constituents of our tested lines to be in accordance with the major chemical constituents reported for grapes [22], watermelon [11] and bottle gourd [12]. In general, the VOC profile reported for grapes and watermelon was constitution of aldehydes such as 2-nonenal, (E,Z)-2,6-nonadienal, alcohols such as 1-hexanol, (E)-3-hexanol, and (Z)-3-hexanol and monoterpenoids [11,17]. In our study, our bottle gourd accessions showed appearance of E-2-nonenal, (E,Z)-2,6-nonadienal and aliphatic alcohols such as 1-hexanol along with three prominent monoterpenoids (Table 1, Figure 5). Therefore, the overall VOC profile of our tested accessions is also in accordance with the VOCs identified earlier in various plant types. Additionally, Chatterjee et al., showed the aliphatic aldehydes to dominate the VOC profile in bottle gourd fruit, our results also confirm the aliphatic aldehydes to be major constituent of the VOC profile among our tested accessions.

The suitability of any plant type for flavor, aroma, and its use several in diagnostic research and health care industry, such as cancer research [23], plant diseases [24] and food industry [25,26] are largely dependent on the volatile compounds occurring in the plant. Therefore, we profiled the VOCs in our bottle gourd accessions to determine the suitability of our accessions to be used in the agriculture sector, health care industry and for domestic uses.

### 4.1. Domestic Use of Identified VOCs and Bottle Gourd Accessions

Among the three identified monoterpenoids in our tested bottle gourd accessions, 1,8-cineol commonly known as eucalyptus oil, upregulated in USVL10CYN is a widely used flavoring agent in oral hygiene products [27]. Apart from this, it is also used in homes to repel insects and thus has important use in the industry to develop insect-resistant treatments [27]. It has strong antimicrobial and antioxidant properties which makes it an excellent food packaging material for ground beef packaged products [28]. Similarly, D-limonene is another important monoterpenoids, (upregulated in USVL10A), identified among our accessions is a commonly found terpenes in nature. It is a major constituent in several citrus oils (orange, lemon, mandarin, lime, and grapefruit) [24,25,26]. D-limonene is listed in the code of federal regulations as generally recognized as safe (GRAS) flavoring agent and can be found in common food items such as fruit juices, soft drinks, baked goods, ice cream, and pudding [26]. It is widely used as a degreaser, cleaning agents for metals, in the electronics industry and in the printing industry in various proportions [29]. On the other hand, hexanal is an aliphatic aldehyde, upregulated in the line USVL10A, and USVL10B is a colorless liquid with a fruity odor. It is used as a flavoring additive, as an insecticide, and is also widely used in the industry to prepare antiseptics, perfumes, plasticizers, and other chemicals for domestic use [30].

### 4.2. Potential Use of Bottle Gourd Accessions with Respect to VOC Profile in Health Care Industry

Aliphatic aldehydes, being major contributors of the VOC profile among our tested accessions consists of compounds with important role in health care industry. Nonanal, also known as a C9 aldehyde, is a saturated fatty aldehyde which is involved in cancer studies and diagnostics and can be studied further to be potentially useful to treat cancer. It has a role as a human metabolite and a plant metabolite [5,31]. Prominent VOCs belonging to the class of aldehydes are studied to be associated with five different types of cancers in humans. Nonanal has also been reported to be used as cancer biomarker for several types of cancers in humans [32]. Upregulation of nonanal in USVL10A and USVL10B suggests the potential of respective accessions to be deployed for human disease diagnostic and research studies. Experiments can be conducted in future to identify the potential use of extracted aldehydes from bottle gourd fruit to be deployed in cancer diagnostics. Similarly, the aliphatic alkane, 2-methoxy-2-methylpropane (upregulated in USVL10A and USVL10B) is also helpful to treat gallstones [33]. Because of its gastric acid neutralizing effect and its support of normal peristalsis, it has also been used for relief of heartburn and gastroesophageal reflux (GERD). D-limonene along with its potential domestic use, has been studied to play important role in chemo preventive activity against many types of cancer [33]. Evidence from a phase I clinical trial demonstrated a partial response in a patient with breast cancer and stable disease management for more than six months in three patients with colorectal cancer [34]. Similarly, α-pinene (upregulated in USVL10A) is a well-known representative of the monoterpenes group and found in many plants derived essential oils [27]. The monoterpenoid, 1,8-cineol is also a natural plant-derived healing agent and has mucolytic, anti-inflammatory properties due to which is widely used to treat inflammatory diseases [35]. A wide range of pharmacological activities have been reported, including antibiotic resistance modulation, anticoagulant, antitumor, antimicrobial, antimalarial, antioxidant, anti-inflammatory, anti-leishmania, and analgesic effects [27]. 

Benzaldehyde an aromatic compound is well known for its anti-cancer effects, first reported in 1980 in Japan. A total of ninety patients with terminal stage cancer and other types of tumors were orally and rectally administered with benzaldehyde (beta-cyclodextrin benzaldehyde inclusion compound (CDBA)) with four doses. Nineteen patients responded completely to the treatment while ten patients responded partially. Thus, further studies were needed to address the suitability of benzaldehyde for cancer treatment. Following the Japan study, recent study conducted by Saitoh et al., (2016) confirmed the use of benzaldehyde as an anti-cancer agent showed that Benzaldehyde inhibits major signaling pathways in the activated cancer cells in pancreatic cancer [36]. Therefore, the identified VOCs in our tested bottle gourd lines can be used to study and explored to be used in health care sector to improve treatment to various ailments.

### 4.3. Suitability of Tested Bottle Gourd Accessions as Important Modulators of Plant Health and Agriculture

Apart from human health care uses, the use of VOCs has effectively been used in agriculture to tackle climate change issues and to ensure food availability to the growing world population. VOCs have prominently been involved as biological control of plant diseases, for management for biotic and abiotic stresses [37], pre and post-harvest treatments of food and crops in recent years. 3-methyl-3-buten-1-ol- is an aliphatic alcohol, upregulated in USVL10A and USVL10B and found in moderate amount in USVL5 (Figure 3), is a secondary metabolite and has been shown to have an important role in plant defense and signaling mechanism. Among the identified Aliphatic aldehydes, Z)-3-hexenal is popularly explored for its defensive role [38] in tritrophic interactions (Z)-3-hexenal has shown important role in plant-to-plant communication, and referred to as the priming effect of volatiles [39]. Recently, Jin et al., (2023) showed that (Z)-3-hexenal appeared to be general response signals triggered by several biotic and abiotic stress signals in plants, therefore it can be explored further for biotic and abiotic stress management in plants [40]. Moreover, the airborne (Z)-3-hexenol (upregulated in USVL10A) from wounded plants trigger the pre-defense responses of neighboring uninfected plants as a defense against the pathogen attack [39,41]. Similarly, hexanal (upregulated in USVL10A), a C-6 aldehyde has been implicated to have antimicrobial properties. A study was conducted to determine the antifungal activities of hexanal vapor against major postharvest pathogens of banana viz., *Colletotrichum gloeosporioides* and *Lasiodiplodia theobromae* [31]. Postharvest treatment of banana with hexanal vapor resulted in phospholipase D inhibition and cell wall thickening of the treated fruit through impeding the penetration of the pathogenic spores [31,32]. The defense-related protein intermediaries increased in hexanal vapor-treated banana fruit, which suggests induced-resistance against *C. gloeosporioides* and *L. theobromae*, via the phenylpropanoid pathway, which plays a significant role in hindering the pathogen quiescence. It also is involved with enhancing the shelf life of bananas [31]. Benzaldehyde (upregulated in USVL10A), the simplest aromatic aldehyde found in our data, is one of the most wide-spread volatiles that serves as a flavor, pollinator attractant [33], and antifungal compound [34,35]. Gao et al., 2018 observed a positive correlation between the level of benzaldehyde and the inhibition of *M. fructicola* in vitro [36] and was found effective to control the ring rot disease of potato caused by *Clavibacter michiganensis* subsp. *Sepedonicus* [37]. The soil application of benzaldehyde on black scurf disease of potato plants also revealed significant reduction of the growth of the causal agent, *Rhizoctonia solani* [42]. Therefore, there is a potential of using benzaldehyde for treatment of various fungal diseases. A potential future insight could be exploring its use for treatment of viral diseases in various plant species.

Based on the VOC profile, we speculate the USVL10A and USVL10B can be deployed for improving plant traits such as plant defense response, as well as for medical purposes. More specifically, USVL10A appears to be consistent of highest abundance for major VOCs and seems to be the most suitable candidate to be explored more for agriculture sector, plant resistance, human diagnostic and health care sector, research studies and food industry. However, the USVL5 can be used for culinary purposes with a moderate number of VOCs.

Since USVL10A, USVL10B and USVLCYN are derivatives of the same line, the properties are very much similar and can be used in the breeding program to attain homozygous accessions that are useful for the plant disease resistance studies.

## 5. Conclusions

Our study evaluated the VOC profile in the two bottle gourd accessions and the derivatives. A total of 60 VOCs were identified. Our results indicate that the bottle gourd profile is dominated by the appearance of aliphatic aldehydes in general. Among our tested accessions, USVL10A was enriched with highest proportion of in hexanal, nonanal, 2-methoxy-2-methylpropane, D-limonene, α-pinene, 3-methyl-3-buten-1-ol-, (z)-3-hexenal, hexanal, benzaldehyde, 2,4-dimethyl-heptane and 1,8-cineol. Based on the function of identified VOCs and their appearance in the tested accessions, USVL10A seems to be a promising candidate for domestic use, plant health industry, agriculture, and health care industry. This line can be further explored to be incorporated in breeding programs for improving the agriculture sector with respect to plant disease management, human health care industry and for various domestic uses.

## Figures and Tables

**Table 1:** The percentage composition of volatile organic compounds in tested bottle gourd accessions, extracted via solid-phase microextraction (SPME) and abundance determined via GC-MS.

Compound	RT	RI	Abundance (Total Ion Current (×10^6^))			
			USVL10CYN	USVL5	USVL10A	USVL10B
**Aliphatic alcohols**						
Ethanol	9.304	480	3.85	0.88	1.92	4.98
2-Methyl-2-propanol	11.056	532	7.50	0.89	4.03	2.61
2-Methyl-1-propanol	14.389	623	0.80	0.00	0.00	0.00
1-Penten-3-ol	16.741	683	2.18	5.71	4.50	3.26
3-Methyl-3-buten-1-ol	18.989	737	0.87	0.91	3.86	3.91
2-Methyl-1-butanol	19.196	742	0.00	0.61	0.75	0.76
(Z)-2-Penten-1-ol	20.410	771	1.22	1.30	1.65	1.27
3-Methyl-2-buten-1-ol	20.799	780	0.00	0.00	0.53	0.82
(E)-2-Hexen-1-ol	25.006	874	0.00	0.00	0.84	0.74
1-Hexanol	25.120	877	2.15	3.38	6.47	5.81
2-Methylbutan-2-ol	15.071	641	3.63	0.75	3.00	2.35
2-Ethyl-1-hexanol	32.299	1038	0.57	0.52	1.00	0.78
**Aliphatic aldehydes**						
Decanal	12.093	561	1.35	1.25	1.59	1.60
3-Methyl-butanal	15.740	658	1.18	13.77	17.17	16.48
2-Methyl-butanal	16.139	668	0.65	10.90	15.12	13.53
Pentanal	17.459	701	4.28	6.37	3.46	4.94
(Z)-3-Hexenal	22.025	808	1.38	2.99	9.95	4.94
Hexanal	22.178	811	118.94	117.85	381.07	247.74
(E)-2-Hexenal	24.713	868	98.21	204.48	412.63	295.18
(E,E)-2,4-Hexadienal	27.435	928	1.75	5.86	22.03	10.62
Octanal	31.346	1016	0.67	0.69	1.13	0.92
Nonanal	35.566	1115	4.71	5.68	9.85	9.99
(E,E)-2,6-Nonadienal	37.641	1167	0.81	0.84	1.92	1.87
2-Methyl-propanal	37.865	1173	0.61	3.18	3.27	3.52
(E)-2-Nonenal	39.522	1217	2.81	1.94	3.48	3.73
**Aliphatic ketones**						
Acetone	10.145	505	86.07	54.72	47.35	97.93
1-Penten-3-one	16.877	686	1.25	5.66	4.54	2.95
2,3-Pentanedione	17.187	694	0.00	0.25	0.71	0.00
4-Methyl-2-heptanone	28.398	950	0.55	0.00	1.26	0.60
6-Methyl-5-hepten-2-one	30.447	995	1.25	1.11	2.49	1.45
**Amines**						
9-Phenanthrenamine	33.833	1074	1.59	1.27	2.09	1.15
N-Methylallylamine	34.643	1093	0.43	0.00	0.91	0.00
**Aliphatic alkanes**						
3,3-Dimethyl-octane	31.515	1020	0.67	0.00	0.95	0.00
Propyl-cyclopentane	34.469	1089	0.32	0.00	0.79	0.00
2-Methoxy-2-methyl-propane	12.394	569	13.11	6.06	21.26	21.67
2-Ethoxy-2-methyl-propane	14.157	617	0.39	0.00	6.02	0.72
3,3,4-Trimethyl-decane	20.099	763	0.00	0.74	2.87	1.11
4-Methyl-heptane	20.656	776	0.00	0.00	3.02	0.41
3-Methylheptane	20.958	783	0.00	0.00	2.03	0.00
2,3-Dimethyl-heptane	22.967	829	0.00	0.00	0.66	0.00
2,4-Dimethyl-heptane	23.206	834	0.00	0.00	2.11	1.00
3,3,5-Trimethyl-heptane	31.693	1024	0.72	0.00	0.98	0.00
2,2,5-Trimethyl-hexane	21.372	793	0.32	0.00	2.40	0.51
**Aliphatic alkenes**						
1-Nonene	33.990	1077	0.71	0.94	2.14	1.92
2,4-Dimethyl-1-heptene	24.135	855	0.97	0.00	6.08	0.93
**Aromatic compounds**						
Benzaldehyde	30.22	990	2.6316855	4.1705	4.696644	4.594236
Benzeneacetaldehyde	33.63	1069	0	4.1738	10.63422	7.944508
1-Ethyl-2,3-dimethyl-benzene	32.82	1050	0.5095425	0.574	0.700705	0
Toluene	21.09	786	0.22938	0	0	0.719768
**Monoterpenoids**						
1,8-Cineol	33.41	1064	0.702644	0	0	0
D-Limonene	33.08	1056	21.92807233	21.856	31.48846	20.5272
α-Pinene	29.06	964	0.455233	0.7263	1.18271	0.822722
**Furans**						
2-Methylfuran	14.01	613	1.469667333	0.2473	0	0
Tetrahydro-furan	14.71	632	0.5224715	0.3427	0	0
2-Pentyl-furan	30.91	1006	1.039926	0.334	1.245199	0
2-Ethylfuran	17.64	705	0	1.1901	1.595573	1.352609
**Others**						
2-Methoxy-3-(1-methylpropyl)-pyrazine	38.191	1182	0.00	0.00	0.96	0.59
Methoxy-phenyl-oxime	25.235	879	31.83	25.39	0.00	46.81
Ethyl 2-cyanocrotonate	30.024	986	0.00	0.58	0.00	0.00
2,3,6-Trimethyl-carbazole	27.665	933	5.85	7.62	13.55	6.88

## Data Availability

The data is presented in tables and figures in this study and are available on request from the corresponding author.
